# Preparation and biomedical applications of artificial cells

**DOI:** 10.1016/j.mtbio.2023.100877

**Published:** 2023-11-24

**Authors:** Qian Xu, Zeping Zhang, Pauline Po Yee Lui, Liang Lu, Xiaowu Li, Xing Zhang

**Affiliations:** a Department of Materials Physics and Chemistry, School of Materials Science and Engineering, Key Laboratory for Anisotropy and Texture of Materials, Ministry of Education, Northeastern University, Shenyang, Liaoning, 110819, China; b Institute of Metal Research, Chinese Academy of Sciences, Shenyang, Liaoning, 110016, China; c School of Materials Science and Engineering, University of Science and Technology of China, Hefei, Anhui, 230026, China; d Department of Orthopaedics and Traumatology, The Chinese University of Hong Kong, 999077, Hong Kong

**Keywords:** Artificial cells, Bottom-up approach, Microcapsules, Cellular communication, Bioreactors

## Abstract

Artificial cells have received much attention in recent years as cell mimics with typical biological functions that can be adapted for therapeutic and diagnostic applications, as well as having an unlimited supply. Although remarkable progress has been made to construct complex multifunctional artificial cells, there are still significant differences between artificial cells and natural cells. It is therefore important to understand the techniques and challenges for the fabrication of artificial cells and their applications for further technological advancement. The key concepts of top-down and bottom-up methods for preparing artificial cells are summarized, and the advantages and disadvantages of the bottom-up methods are compared and critically discussed in this review. Potential applications of artificial cells as drug carriers (microcapsules), as signaling regulators for coordinating cellular communication and as bioreactors for biomolecule fabrication, are further discussed. The challenges and future trends for the development of artificial cells simulating the real activities of natural cells are finally described.

## Graphical abstract

**Figure:**
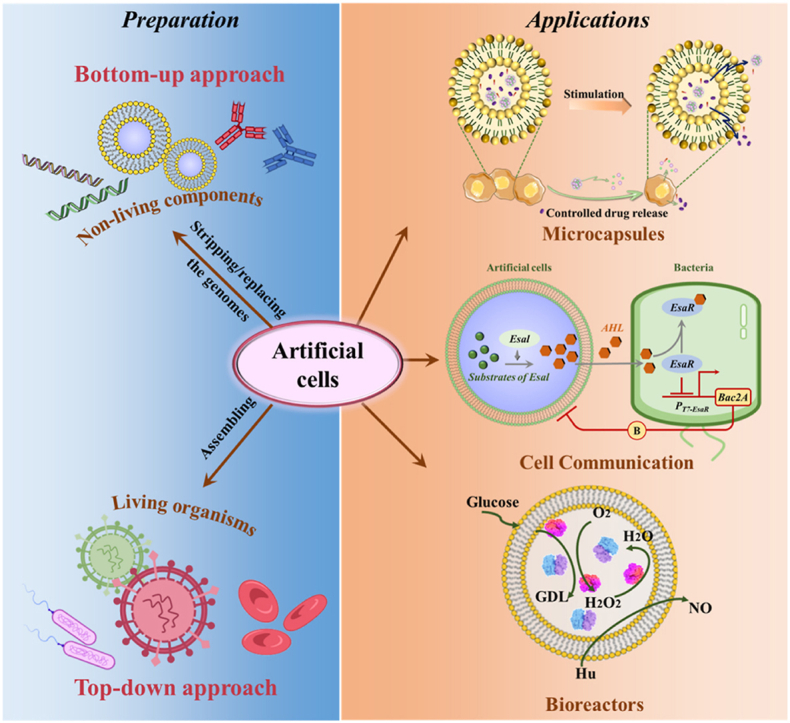

## 1 Introduction

Cells are the basic structural and functional units of living organisms. Cell therapy using various cell types especially stem cells and immune cells has been explored for treatment of human diseases and tissue/organ regeneration [1,2]. For example, human mesenchymal stromal cells have been tested for the treatment of COVID-19 in a pilot clinical study, demonstrating significantly lower mortality in the intervention group compared to the control group (intervention group: 7.69% and control group: 33.33%) [1]. However, there are some limitations of cell therapy including its complex manufacturing process and high production cost. Moreover, the cellular biological activities can be altered during *in vitro* expansion. There is also a shortage of autologous cells from patients. Immuno-rejection may occur if allogenic cells are used for cell therapy. Therefore, the idea of constructing artificial cells to overcome these limitations of natural cells is highly attractive [3,4].

**Fig. 1:**
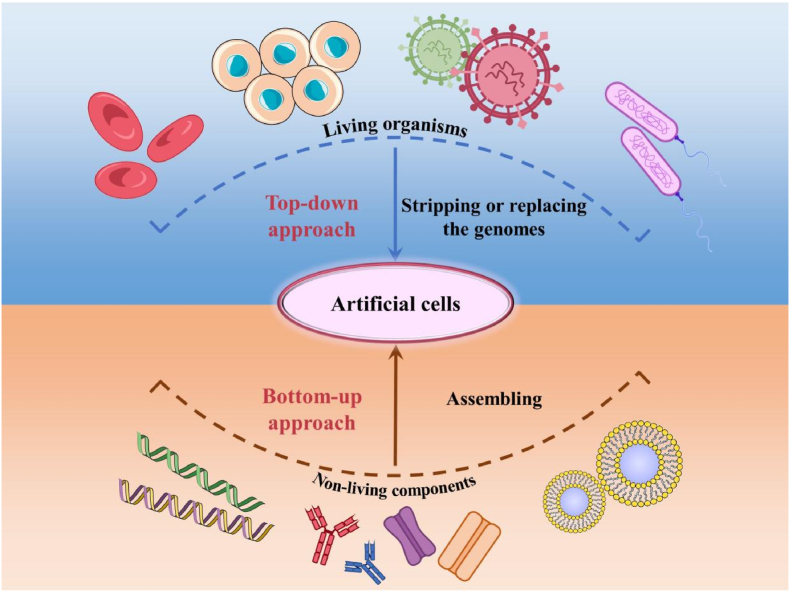
The fabrication of artificial cells using top-down or bottom-up approaches.

“Artificial cell” generally refers to a synthetic cellular system that mimics the properties and functions of natural cells, but does not possess the inherent complexity or organization of natural cells. The concept of artificial cells was first proposed in 1957, which reported the fabrication of artificial red blood cells containing hemoglobin [5]. Since then, researchers have attempted to encapsulate proteins, hormones, enzymes, drugs, and other components in artificial cells [6,7]. Both top-down and bottom-up strategies have been used for the preparation of artificial cells (Fig. 1) [8,9]. The top-down methods seek to reduce the complexity of living cells’ genomes while maintaining their most important features and essential functions [[10], [11], [12]]. However, it is difficult to remove genes one at a time or completely replace the whole genome of existing organisms, thereby limiting the practical applications of top-down methods. On the other hand, the bottom-up methods put together non-living and purified active components to assemble artificial cells in order to obtain essential characteristics of natural cells [13,14].

Artificial cells can imitate various functions of natural cells, such as growth [15], membrane fusion [16], protein synthesis [17], self-replication of DNA [18], transcription and translation [19], and operate biochemical devices with liposomes [20,21]. The first sustainable self-replicating *M. mycoides* cells with a synthetic genome were created in 2010 [22]. A de novo membrane self-assembly method for the preparation of artificial cells was then designed via a biomimetic coupling reaction [23]. In 2015, researchers investigated the preparation and growth of phospholipid membrane of artificial cells driven by a self-reproducing catalyst [24]. Moreover, artificial cells containing a nucleus-like DNA-hydrogel compartment were fabricated, which could express proteins and communicated with the neighboring artificial cells through the diffusion of protein signals, coordinated quorum sensing (QS) and underwent cell differentiation like natural cells [25]. Artificial cells could also be fabricated as a bioreactor for DNA/RNA replication, protein synthesis and the substance from extracellular matrix microenvironment [[26], [27], [28], [29], [30]]. For example, artificial cells containing polymerase chain reaction (PCR) reagents were designed for DNA amplification [31]. In addition, artificial cells were reported to show potential for the treatment of various diseases like myocardial infraction and cancer [32,33].

As a result of the significant progresses made in the research of artificial cells in recent years, this review aims to summarize the advantages and limitations of different fabrication techniques, and discuss the applications of artificial cells as drug carriers (microcapsules), as signaling regulators for coordinating cellular communication and as bioreactors for biomolecule fabrication. Depending on the specific goals of the research, artificial cells can range from simple lipid nanoparticles to more complex systems containing multiple biomolecules and molecular machineries. In this review, we would take a board definition of artificial cells, which encompass a diverse range of structures and systems.

## 2 Preparation of artificial cells

The top-down approach and the bottom-up approach are two complementary approaches for fabricating artificial cells [8,[34], [35], [36]]. The top-down approach has been used to sequentially delete superfluous genes in a natural cell, or replace the original genome entirely with a synthetic genome, while still performing specific biological functions [22,[37], [38], [39]]. The bottom-up approach, on the other hand, builds artificial cells with cell-like structures and functions by assembling non-living natural or artificial molecular substances [[40], [41], [42]]. However, the construction of artificial cells using a top-down approach remains a significant challenge due to the complex genetic composition of natural cells. In this review, we primarily focus on the bottom-up approaches, which enable simpler, faster, and large-scale production of artificial cells.

### 2.1 Amphiphilic phospholipids/polymers vesicle-based artificial cells

For the bottom-up approach, artificial cells constructed in various forms (in tubes or micro-scale chambers) are composed of selectively permeable membranes similar to natural cells that can separate the cell interior from the extracellular environment [43]. The materials for preparing the membranes of artificial cells include amphiphilic phospholipids [[44], [45], [46]], amphiphilic polymers [47], a mixed composition of phospholipids, block copolymers [48,49], and cell membrane-derived materials [50,51] that can regulate the permeability of artificial cells.

**Fig. 2:**
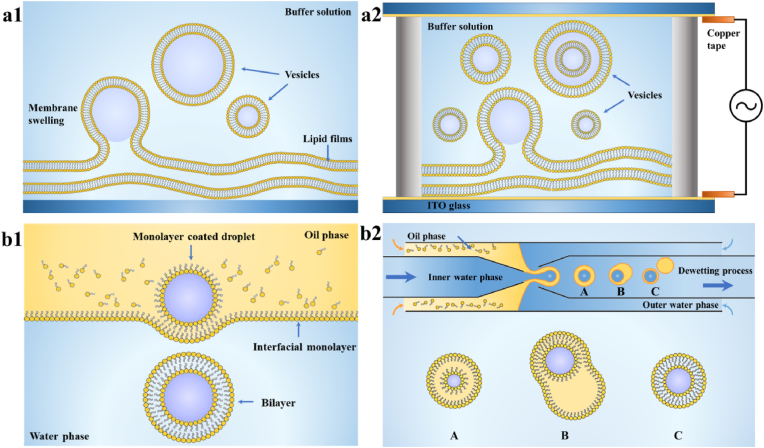
Common bottom-up methods for the fabrication of artificial cells. (a) Thin-film hydration strategy: (a1) gentle hydration method, (a2) electro-formation method. (b) Emulsion templating strategies: (b1) water/oil (W/O) emulsion transfer method, (b2) water/oil/water (W/O/W) double emulsion template method.

The thin-film hydration strategy and emulsion templating strategy are most commonly used in the bottom-up approach for the fabrication of amphiphilic phospholipids/polymer-based artificial cells (Fig. 2). The thin-film hydration strategy is the classical method to fabricate artificial cells, and the organic solvents are used to form the thin film of amphiphiles. Subsequently, an aqueous solution is then added to the dry thin film to form the liposomes. Although the thin-film hydration strategy is simple to operate, the vesicles exhibit variable sizes and low encapsulation efficiency [8,42]. Therefore, the emulsion templating strategy has been developed to overcome the limitations of thin-film hydration strategy. The size and shape of vesicles can be tailored by controlling parameters such as amphiphilic compositions, flow rate, and the diameter of capillary.

#### 2.1.1 Thin-film hydration strategy

The gentle hydration method has been used to prepare the chambers of artificial cells based on the thin-film hydration strategy for long time. In this method, the phospholipid membrane is uniformly deposited on the glass surface, and the phospholipid vesicles are then spontaneously formed via the hydration of the phospholipids after the addition of water or the buffer solution. The process is driven by the flow of the aqueous solution between the bilayers of the phospholipid membrane. However, the hydration process produces a large number of multilamellar vesicles and lipid debris, resulting in the relatively poor quality of phospholipid vesicles [52,53]. Furthermore, the encapsulation efficiency of active substance within phospholipid vesicles is relatively low [42]. In addition, the preparation process is time-consuming with a relatively low yield, which is inevitable to prepare both monolayers and multilamellar phospholipid vesicles at the same time [54].

The application of an alternating current proposed by Anglova can promote the hydration and swelling of phospholipid membrane [14,53]. The phospholipid membrane layer should be uniformly deposited on special electrode surfaces, such as Pt wire [55], indium tin oxide (ITO) electrode [56,57] and stainless steel electrode [58]. Giant unilamellar vesicles (GUVs) with diameters in the range of 5–200 μm were prepared using an alternating current (frequency: 10 Hz; voltage of 1–7 V; time: 2 h) produced by the electrodes submerged in an aqueous solution [59]. However, the applied alternating current can peroxidize lipid acyl chains, thus changing the phospholipid composition and properties of the vesicles during electro-formation [59]. For example, the lyso-compound (one of the byproducts of lipid peroxidation) formed after electro-formation with one or multiple cleaved fatty acid chains showed high solubility in the aqueous solution [59,60]. The area expansion modulus in egg phosphatidylcholine (EPC) vesicles was reduced from 171 mN m^−1^ to 82 mN m^−1^ by adding 30 mol.% monooleoylphosphatidylcholine inside EPC vesicles [60]. Similar to the vesicles formed by the gentle hydration method, the GUVs formed by the electro-formation method showed a large size distribution.

**Fig. 3:**
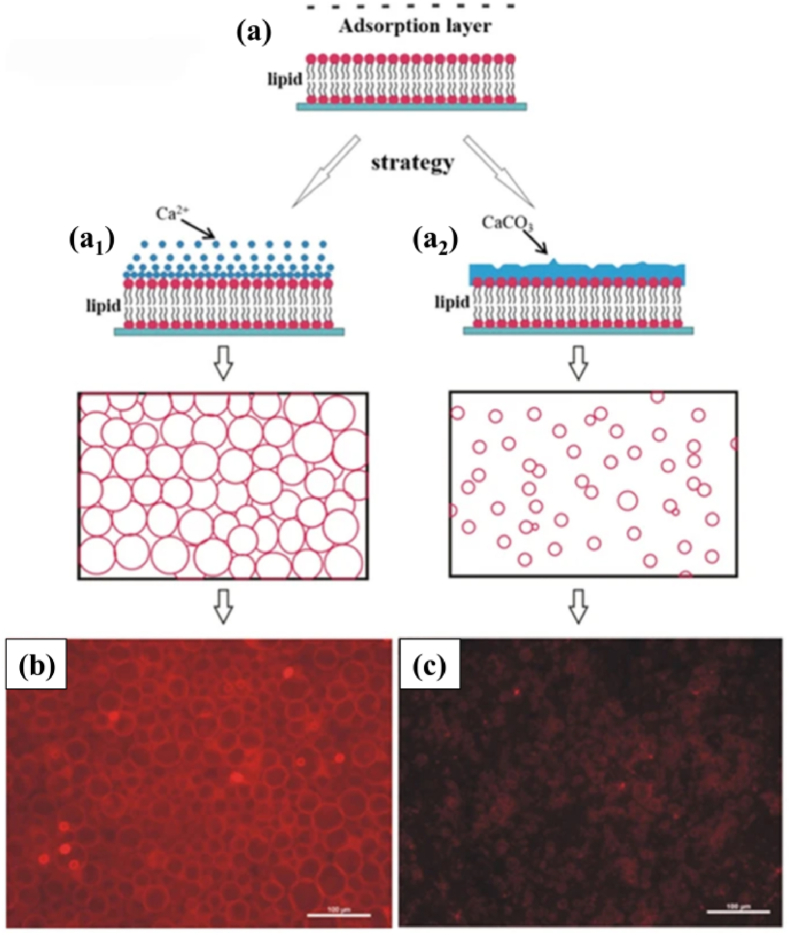
The calcium absorption approaches for forming GUVs of uniform size. (a) Schematic diagram of the two approaches: (a_1_) the ions adsorption layer method and (a_2_) the mineral adsorption layer method. (b) Fluorescent GUVs synthesized by method (a_1_). (c) The fluorescent GUVs layer prepared by method (a_2_) [64].

Many studies attempted to prepare vesicles in similar sizes [56,[61], [62], [63], [64]]. Han et al. developed the novel ITO electrodes containing coplanar interdigitated array fabricated by the combination of photolithography and electrochemistry methods, leading to the formation of phospholipid vesicles of similar sizes [56]. The sizes of the phospholipid vesicles were controlled by the electrode width. With the increase of the electrode width, the field strength decreased, resulting in the smaller vesicles [56,61]. Besides, GUVs (with the diameters of ∼10–30 μm) can be efficiently purified by centrifugation or membrane filtration within 10 min [62,63], and the fraction of vesicles less than 10 μm deceased from 13% (±1%) to 4% (±1%). In addition, phospholipid vesicles with uniform size can be obtained by in situ calcium absorption approach by applying calcium-containing compounds (calcium ions (Ca^2+^) and calcium carbonate minerals (CaCO_3_)) on the surface of phospholipid membranes to limit the fusion and growth of GUVs [64]. As shown in Fig. 3a_1_, a Ca^2+^ adsorption layer under an alternating current was transformed into a patterned Ca^2+^ “barrier” with a narrow size range (30 ± 8 μm) (Fig. 3a_1_). The mineral adsorption layer prepared by in situ CaCO_3_ formation around the phospholipid layer can also form GUVs (Fig. 3a_2_) (10 ± 3 μm). Therefore, the common problems such as the difficulty to control electro-formation of GUVs at a high voltage and the risk of encapsulating electro-sensitive molecules in GUVs during electro-formation can be resolved.

In summary, the electro-forming method enables the control of experimental parameters (such as voltage, frequency, and time) and allows easier and quicker preparation of monolayer phospholipid vesicles with uniform size compared to the gentle hydration method. However, some encapsulated biomolecules can be oxidized due to the application of an electric field, which restricts the application of the electro-formation method [42].

#### 2.1.2 Emulsion templating strategies

The water/oil (W/O) emulsion transfer method was first reported by Weitz for the preparation of phospholipid vesicles in 2003 [65,66]. A W/O emulsion was prepared by vortexing phospholipid containing mineral oil or decane solution, transferring it to a buffer solution, then passing through the oil-water interface to form vesicles. In order to obtain unilamellar liposomes with better-controlled features, the W/O emulsion transfer method was often combined with microfluidic devices to prepare phospholipid vesicles. Malmstadt et al. proposed a microfluidic technique using a two-step method to fabricate compositionally asymmetric GUVs [67]. First, a monolayer of oil droplets was formed in a microfluidic device. The droplets were then passed through an oil-water interface to form phospholipid vesicles with a monolayer. Paegel et al. integrated a microfluidic assembly line containing both emulsions and phospholipid vesicles in one microfluidic device to produce vesicles [68]. Uniform and stable lipid droplets with an aqueous interior in an oil phase were produced by the convergence of the water and oil phases. The droplets impinged on a triangular post and passed through the water and oil interface to form unilamellar vesicles [68].

To ensure the precise control and mass production of phospholipid vesicles, a water/oil/water (W/O/W) double emulsion template method was also developed by microfluidic devices based on the W/O emulsion transfer method [69,70]. The vesicles were prepared by forming a W/O/W emulsion, followed by the removal of the organic solvent through the dewetting process [[70], [71], [72]]. The interfacial tension of vesicles can be adjusted by modulating the W/O/W emulsion components [71,[73], [74], [75]].

A double emulsion comprising an aqueous inner phase, an oil phase of 1-octanol to dissolve phospholipids, and an aqueous outer phase containing 15% (v/v) glycerol was constructed in the previous studies [[74], [75], [76]]. Interestingly, 1-octanol droplets could be fully separated from the assembled liposomes within a few minutes. This phenomenon was due to the higher interfacial tension between 1-octanol and water (8.5 mN m^−1^) than the membrane tension of liposomes (only about several μN·m^−1^). The short 1-octanol molecules formed defects in the ordered structure with longer lipid molecules. These factors together led to the rapid and efficient separation of 1-octanol molecules and lipid molecules, which induced self-assembly of lipid bilayers along the interface of the double-emulsion droplets, resulting in vesicles with a size of about 5–20 μm. In addition, these unilamellar vesicles could be again injected into the microfluidic device as internal phases to provide larger liposome-loaded W/O/W emulsion droplets that undergo a second dewetting transition, resulting in multicompartment vesicles or more complex concentric multicompartment vesicles [71,72].

The combination of emulsion templating strategy with microfluidic devices for the preparation of phospholipid vesicles have the advantages of high yield, uniform and controllable vesicle size, less residual solvent, as well as controllable structure. However, this method results in a mixed solution of vesicles, organic droplets and other residues, which need to be further purified. Tivony et al. developed a microfluidic platform based on pinched-flow fractionation to form two bifurcated streams: one containing all residues and the other consisting of vesicles in a residue-free solution [77]. These two streams were divided into five microchannels for the collection of purified vesicles with high efficiency (e = 0.99) based on their size. Additionally, Yandrapalli et al. designed a high-throughput PDMS microfluidic device using a double cross-junction structure to form lipid vesicles free of surfactants and additives [78].

#### 2.1.3 The combination of top-down and bottom-up approaches

Currently, the artificial cells produced by top-down or bottom-up approaches cannot match the complexity of their biological counterparts. Many researchers have tried to combine the top-down and bottom-up approaches for preparing the hybrid living-synthetic system with the advantages of both natural and artificial cells [36,[79], [80], [81], [82], [83], [84], [85], [86], [87]].

**Fig. 4:**
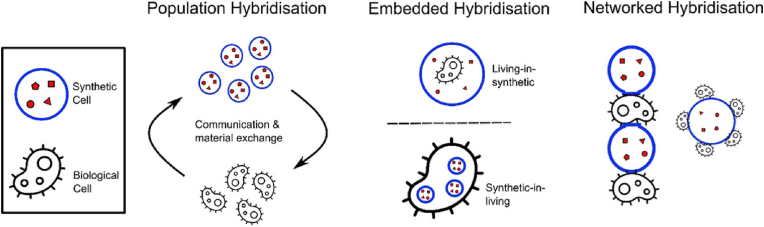
Schematic of the three different cellular bionic hybridisation modes [36].

Fig. 4 illustrated three potential hybrid approaches [36]. The first approach involved population hybridisation, which the biological and artificial cells interacted and exchanged information in a spatial manner [[79], [80], [81]]. The second approach was embedded hybridisation, which entailed the encapsulation of living cells within synthetic cells or vice versa [[82], [83], [84], [85]]. Lastly, networked hybridisation involved the physical connection of artificial and biological cells in a network or tissue-like structure while maintaining their identities [86,87]. The hybrid cellular systems was an emerging field with strategic significance in bioengineering, driving innovation and expanding the applications of synthetic biology [36].

### 2.2 Hydrogel-based artificial cells

Artificial cells prepared by amphiphilic phospholipids/polymers possess an internal aqueous compartment that enables the execution of a variety of biochemical reactions in a cell-like manner including enzyme catalysis, DNA amplification, and gene expression [88,89]. However, these processes are often restricted by the permeability of the membrane, which produce an obstruction to these reactions. Furthermore, these artificial cells exhibit poor stability and are sensitive to environmental factors such as pH value and temperature, thereby limiting their practical applications [89]. In contrast to the aqueous compartment commonly used in artificial cell systems, hydrogels offer a promising alternative. Hydrogels are three-dimensional crosslinked polymer networks formed by simple reactions involving one or more monomers [90,91]. Hydrogels exhibit lower molecular diffusion rates, higher viscosities and Young’s moduli, making them more analogous to the internal matrix of natural cells [89,92]. Consequently, hydrogel-based artificial cells have garnered significant attention and have been extensively investigated.

The development of hydrogel-based artificial cells typically involves the preparation of micro- and nanogels [92]. The former often serves as mimics of organelles and cells [93], while the later resembles extracellular vesicles in terms of their size and are commonly employed as substitutes for biological cell signaling media [94]. This approach allows the replication of key cellular processes and functions, providing a more accurate representation of natural cell behavior within the artificial cell systems [92,95]. Microfluidics has emerged as a valuable tool for generating micro- and nanogels to enhance the preparation efficiency and minimize batch-to-batch variation of artificial cells [96,97]. Toprakcioglu et al. used a two-step soft lithography process to integrate micrometer PDMS channels with nanometer PDMS channels, and formed sub micrometer droplets of protein aqueous solutions with adjustable diameters (from 50 nm to 2.5 μm) by microfluidic control [98]. Subsequently, proteins self-assembly formed hydrogel-based artificial cells with fiber-like networks [98]. In addition, Chu et al. used a microfluidic approach based on triple emulsions to design and produce hydrogel microcapsules with complex multilayered core-shell structures [99].

### 2.3 Colloidosome-based artificial cells

The colloidosome (also known as Pickering emulsions “PE”) was initially developed as a type of microcapsule composed of chemically cross-linked amphiphilic nanoparticles [100]. As aforementioned, vesicle-based artificial cells were constructed using self-assembled bilayer membranes, which can be highly sensitive to external factors such as pH and temperature [89], and have limited permeability. In contrast, colloidal particles were typically covalently cross-linked, ensuring greater stability and durability of the membrane under various physicochemical conditions [101]. Colloidosome-based artificial cells were typically formed through PE polymerization [102] or PE crosslinking [103]. This involves the adsorption of amphiphilic nano/micron solids at the interface of two immiscible liquids, resulting in the formation of stable W/O or oil/water (O/W) emulsions [101]. Simultaneously, a single layer of stable material was generated at the interface between the immiscible liquids, creating a robust elastic shell suitable for artificial cells [100]. Li et al. used aqueous buffer microdroplets stabilized by silica (SiO_2_) nanoparticles generated by spontaneous self-assembly of oil-based PE suspensions as artificial cells. Cell growth via methanol production mediated by organosilane subsequently ruptured the inorganic membrane locally, enabling second-generation protocell outgrowth and separation [104]. This colloidosome approach offered a simple and versatile method for producing artificial cells using various liquids, with the ability to control the size of the cells ranging from sub-micron to a few millimeters [35].

## 3 Applications of artificial cells

Artificial cells have potential applications in many fields, such as drug delivery, cell therapy, cell communication, pharmacology, medical diagnostics, bioreactors, sensors, nanocomputers, and agriculture [7,86,[105], [106], [107], [108], [109], [110], [111]]. In this review, we discuss the applications of artificial cells in biomedicine, particularly their role as drug carriers (microcapsules), signaling regulators for facilitating cellular communication, and bioreactors for synthesizing biomolecules.

### 3.1 Artificial cells as microcapsules

Artificial cells exhibit some advantages of immune escape ability and can achieve long-term circulation in blood due to the same surface component derived from the natural cellular membranes [[112], [113], [114], [115]]. Inspired by the prolonged life cycle (∼120 days), high flexibility, and immune escaping function of red blood cells (RBCs), artificial red blood cells (aRBCs) were constructed for systematic long-distance delivery of cancer immunotherapeutic drugs [116]. The aRBCs have both photodynamic function and self-oxygen supply capacity, overcoming the limitation of extreme hypoxia in tumor treatment. For example, artificial cells modified with human-CD47-based peptide exhibited a longer half-life in systemic circulation and an enhanced capacity for targeting and labeling of tumors than those lacking the peptide [117]. Haghgooie et al. fabricated aRBCs with comparable size (diameter: 8 μm; thickness: 2 μm) to natural RBCs by using soft polyethylene glycol (PEG) microparticles functionalized with antibody against epithelial cell adhesion molecule (EpCAM) [118]. These microparticles could pass through thin capillary channels like real RBCs and showed the ability for disease diagnosis and therapy. However, the deformability of aRBCs was not comparable to that of RBCs. The recombinant erythrocyte membrane-derived aRBCs were constructed to encapsulate hemoglobin, polydopamine (as an antioxidant) and polydopamine-carried photosensitizer [119]. The average hemoglobin content (165 pg) in aRBCs was much higher than that of the natural cells (9.2–20.8 pg). Moreover, aRBCs showed higher accumulation in tumor tissue (the fluorescence intensity was about 4 times of that from natural cells), leading to the complete tumor elimination [119].

Artificial cells can also achieve good mobility, penetration and therapeutic effects due to the fusion ability of membranes [32,120,121]. For example, mesenchymal stromal cell (MSC) membrane-coated poly (lactic-co-glycolic acid) particles loaded with the MSC secretion were prepared with a diameter of ∼20 μm. *In vitro* experiment showed that the assembled artificial cells expressed MSC markers (CD105, CD90 and CD4) and secreted growth factors (VEGF, SDF-1 and IGF-1) similar to that of real MSCs [32]. The transplantation of these artificial cells protected heart morphometry in an acute myocardial infarction mouse model, showing therapeutic effects comparable to that of MSCs. Cancer membranes-derived artificial cells can also achieve good therapy effects due to the homologous targeting ability. For example, artificial cells modified by cancer cell-derived histone, DNA and membranes were developed to enhance cancer therapy. In the MCF-7 tumor bearing model, artificial cells exhibited superior gene transfection efficacy than the Lipo group (Lipofectamine™ 2000/DNA complexes) and BC group (DNA/histone complexes). Moreover, artificial cells demonstrated enhanced anticancer effects, with the tumor volume significantly decreased compared to both Lipo and BC groups [112]. The cancer cell membrane-coated silica nanoparticles (SIL) containing photodynamic reagent (Ce6) named as CCM/SIL/Ce6 enhanced the therapeutic effects of photodynamic therapy (PDT) for cancer treatment. The transplantation of these CCM/SIL/Ce6 to the MCF-7 tumor-bearing mice for 14 days down-regulated the expression of CD31 and vascular endothelial-cadherin, and significantly reduced the tumor volume compared to the other groups (SIL/Ce6, Ce6) (tumor volume 52 ± 9 mm^3^ versus 400 mm^3^) [122]. For improved immunotherapy against lung metastasis, researchers designed biomimetic aAPCs (CD-MnOx@CM) using ultrathin MnOx nanoparticles (NPs), which were functionalized with T cell activators (anti-CD3/CD28 mAbs, CD) and tumor cell membranes [123]. The aAPCs enhanced homotypic tumor homing ability and antitumor immune response by activating dendritic cells and CD8^+^ T cells. Moreover, *in vivo* administration of aAPCs could significantly inhibit lung metastasis and extended the survival time of animals, while metastatic nodules invaded more than half of the lung in the untreated group.

**Fig. 5:**
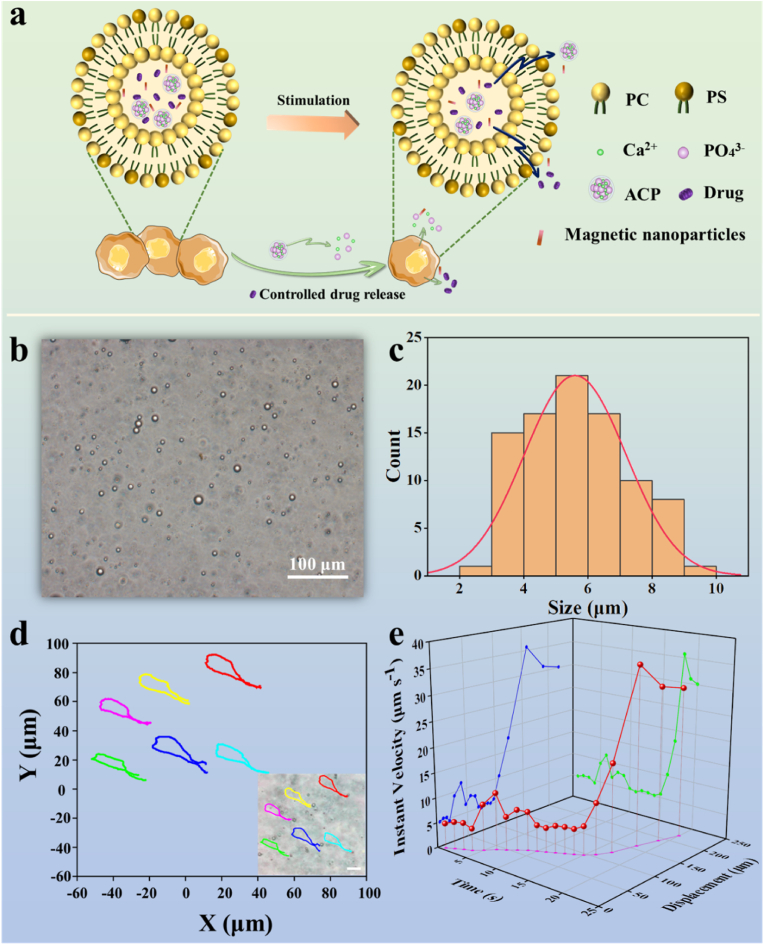
(a) Schematic diagram showing the potential application of artificial cells for enhancing bone formation via controlled release of ACP clusters and drugs upon external stimulation. PC: phosphatidylcholine. PS: phosphatidylserine. (b) The morphology of artificial cells, (c) the particle size distribution of artificial cells, (d) the analysis of cluster motion of artificial cells under the action of a magnetic field, (e) the statistical chart of time, displacement, and instantaneous rate of artificial cells under magnetic drive.

Tang and co-workers created artificial osteoclasts modified with tetracycline (named as TC-OCs) [120]. The migration ability of TC-OCs in tissues was 3-fold higher than that of natural osteoclasts (OCs), and could resorb ectopic bone. OCs and TC-OCs labeled with Calcein AM were injected into the Achilles tendons of the Sprague-Dawley rats at day 4 after tenotomy to further examine the cell viability *in vivo*. The fluorescence intensity of Calcein AM was significantly higher in the TC-OC group (76.6 ± 10.0%) than that of the OC group (27.6 ± 7.7%), suggesting longer survival of TC-OC *in vivo*. The ectopic bone-volume was reduced by more than 70.5 ± 7.6% in the TC-OC group, which was higher than that of the OC group (33.3 ± 7.9%) after 60 days post-injection, indicating superior ability of TC-OC for treating heterotopic ossification [120]. TC-OCs thus might also have the potential for the treatment of other calcified diseases, such as vascular calcification. We recently designed artificial cells (with the diameter 5.60 ± 1.57 μm) for enhancing bone formation, which consisted of a phosphatidylserine (PS)-rich phospholipid membrane bilayer encapsulating amorphous calcium phosphate (ACP) clusters and magnetic nanoparticles (Fig. 5a–c). As shown in Fig. 5d, artificial cells underwent controllable cluster motion under a magnetic field. The instantaneous rate and related displacement of artificial cells within 20 s were evaluated, and the results showed that the instantaneous motion rate could reach 30 μm s^−1^, which was significantly higher that of traditional non-magnetic driven artificial cells. As shown in Fig. 5a, the ACP clusters inside the artificial bone cell could release Ca^2+^ and PO_4_^3−^ ions and promoted biomineralization for enhancing bone formation upon activation by a magnetic field.

In addition, many researchers attempted to achieve better therapeutic effects by adjusting the geometry of artificial cells [124,125]. For example, elliptical artificial antigen presenting cells (aAPCs) with larger contact areas were reported to induce higher expansion and activity of CD8^+^ T cells, compared to the spherical aAPCs. The number of CD8^+^ T cells after treatment with ellipsoidal aAPC was 2-fold of that after treatment with spherical aAPC in a B16 melanoma model, suggesting excellent immune stimulatory capabilities of ellipsoidal aAPC *in vivo* [124]. Although aAPCs showed good immunotherapeutic effects *in vitro*, their functions in the dynamic and complex environment *in vivo* remain unclear. More systematic research is required to examine the impact of aAPCs on the immune system.

### 3.2 Artificial cells as signaling regulators for cellular communication

Artificial cells have been applied to regulate cellular functions by transmitting chemical or biological signals to target cells [126]. The first artificial cells developed for communicating and regulating functions of natural cells used formaldehyde and boric acid as simple raw materials to generate various sugar derivatives, which could induce QS of *Vibrio harveyi* [127]. At present, the signal molecules used in artificial cells for the communication with live cells include signaling molecules on membrane surfaces (e.g., MHC class 1 tetramer, anti-CD28, anti-4-1BB), intracellular substances (e.g., *N*-acyl-homoserine lactones (AHL), homoserine lactones (HSL), glucose, proteins), and nucleic acids (DNA).

**Fig. 6:**
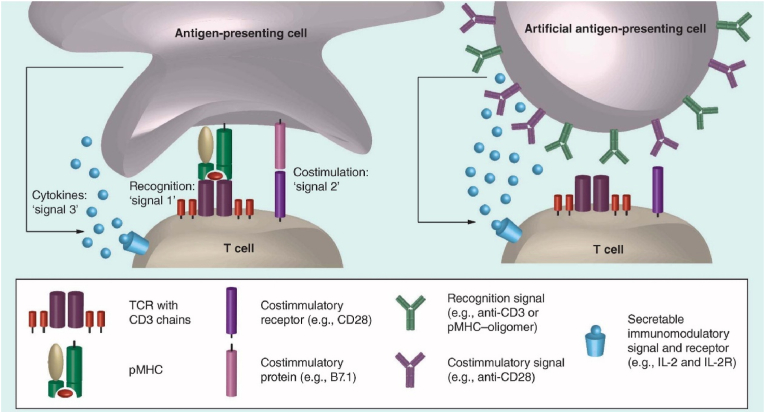
Diagram showing the interaction between T cell and the antigen-presenting cell (APC) or artificial antigen-presenting cell (aAPC) [128].

Communication between artificial cells and natural cells can be achieved by selective interactions among ligands and receptors on cell surfaces. Inspired by the signaling mechanisms adopted by natural cells, researchers simulate information exchange between artificial cells and natural cells by loading or engineering signal molecules onto the surface of artificial cells. For example, to mimic APCs responsible for T cells activation, aAPCs were engineered to present a recognition signal (signal 1, e.g., peptide in MHC (pMHC) oligomer or anti-CD43) and costimulatory signal (signal 2, e.g., anti-CD28) on the surface. Secretable immunostimulatory signal and receptor (signal 3, e.g., IL-2) were engineered in aAPCs to further enhance T cell amplification and differentiation (Fig. 6) [128]. The aAPCs presented with T cell activation ligands were designed for T cell proliferation and activation for cancer immunotherapy [129,130]. Polystyrene aAPCs with surface bounding signal 1 (MHC class 1 tetramer) and signal 2 (anti-CD28 and anti-4-1BB) were reported to inhibit subcutaneous tumor growth as well as delay of tumor progression. aAPCs showed a longer median survival time (MST) (34 days) compared to the anti-CD28/4-1BB group (28 days) and anti-His group (25 days) in B16 melanoma tumor-bearing mice [131]. Moreover, it is much easier to produce aAPCs compared to isolation of natural APCs, thus providing a cost-effective alternative for cellular immunotherapy.

Inspired by the exchange of chemical and biological signals for cell-cell communication, artificial cells were also prepared to produce chemical signals such as QS molecules, organic compounds or DNA for communication with target cells. Simmel’s group established the chemical communication between cell-free systems and bacteria based on two synthetic gene circuits [132]. The artificial cell sender equipped with AHL synthase gene (LuxI) generated AHL, producing a gene expression response in the receiver bacteria controlled by a genetic “AND gate”. Artificial cells encapsulating the gene networks with QS signals were also engineered to kill bacteria by AHL production, achieving the communication between artificial cells and bacteria [133]. AHL was synthesized in artificial cells in the presence of Esal and its substrates. The release of AHL from artificial cells was detected by the bacteria, and then bound to the EsaR to form the AHL-EsaR complex. The bacteria were killed by the expression of Bac2A from the PT7-EsaR promoter and artificial cells were ruptured, resulting in the negative feedback response. Moreover, Mansy and coworkers achieved chemical signal transmission and communication between artificial cells and various bacteria (*V. fischeri*, *V. harveyi*, *E. coli*, and *P. aeruginosa*). The artificial cells could sense and synthesize QS molecules in the presence of HSL [80]. Besides, artificial cells were used to produce chemical signals for communicating and inducing the differentiation of neural stem cells [19]. The pore perfringolysin O (PFO) monomers were synthesized and regulated by both destabilizing the receiver protein (LuxR) and the signaling molecule N-3-oxo-hexanoyl-homoserine lactone (3OC6HSL), then assembled into pores under the cholesterol on the membrane for in situ synthesis and on-demand release of brain-derived neurotrophic factor (BDNF) [134]. In another study, artificial cells containing different types of feedforward and feedback gene circuits were reported to sense the surrounding environment and induce cellular differentiation [135].

**Fig. 7:**
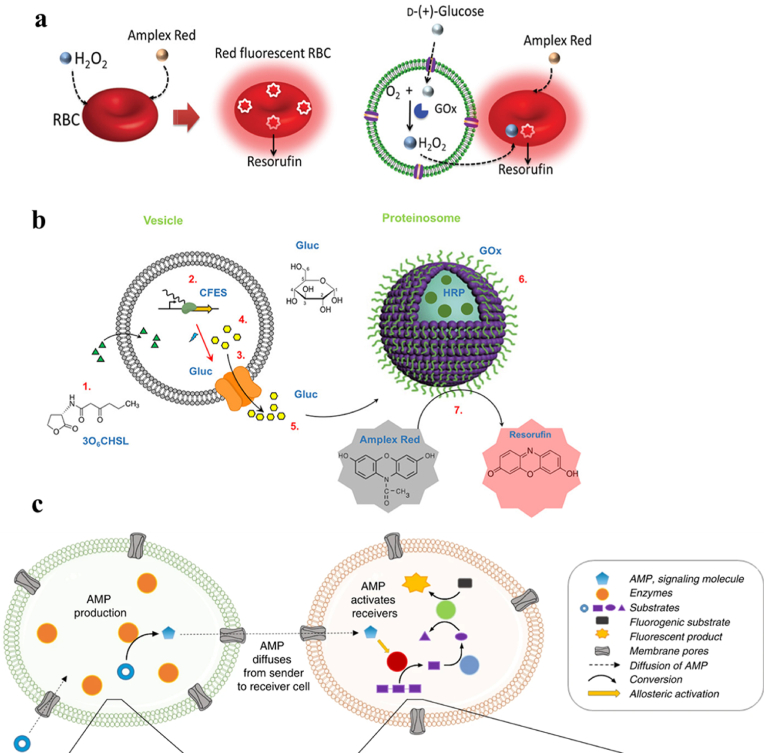
(a) Schematic representation showing that hydrogen peroxide (H_2_O_2_) was generated in artificial cells by the oxidation of glucose in the presence of glucose oxidase (GOx) [136]. (b) A chemical communication pathway between two liposome-based artificial cells (lipid vesicle and proteinosome) [137]. (c) Schematic diagram of signaling molecule (AMP) generated by sender group and spread to receiver group [143].

Chemical communication between artificial cells and natural RBCs was attained through a spatially coupled two-step enzyme cascade reaction [136]. As shown in Fig. 7a, hydrogen peroxide (H_2_O_2_) was generated in artificial cells by the oxidation of glucose in the presence of glucose oxidase (GOx), which was found to be successfully taken up by RBCs. Red fluorescence was then attained by the conversion of amplex red to resorufin. Tang et al. assembled a chemical communication pathway between two liposome-based artificial cells (lipid vesicle and proteinosome). The lipid vesicle containing 3OC6HSL activated the expression of α-hemolysin and the formation of membrane pore, thus triggered glucose release in artificial cells encapsulated cell-free gene expression system (CFES). The hydrogen peroxide generated due to the glucose oxidation occurring on the proteinosome membrane, then reacted with encapsulated horseradish peroxidase (HRP) to convert Amplex Red into a fluorescent output (Fig. 7b) [137]. Some other researchers also reported the cellular signaling between artificial cells by the GOx/HRP cascade reaction [[138], [139], [140]].

Communication between artificial cells can also be designed and controlled by DNA nanostructures [141,142]. Artificial cells (as stimulators) modified with the DNA triangular prism B were designed to stimulate another artificial cells (as receptors) modified with the DNA triangular prism A for DNA messenger release, thus activating synthetic transmembrane channels and the influx of Ca^2+^ [141]. Moreover, long-distance communication between artificial cells was achieved by signal amplification step [143]. As shown in Fig. 7c, sender artificial cells loaded with the enzyme apyrase produced adenosine 5’-monophosphate (AMP) by the sequential hydrolysis of adenosine triphosphate (ATP) with the enzyme apyrase, and then AMP diffused to the receiver artificial cells. The receivers amplified the signal and produced nicotinamide adenine dinucleotide (NADH) by reconstituting a metabolic pathway through adding the downstream enzymes (glucose-6-phosphate dehydrogenase and phosphoglucomutase) [143]. Porous artificial cells containing nucleus-like clay-DNA hydrogels were fabricated. Artificial cells could communicate with each other through releasing protein signals (T3 RNA polymerase) that activated the gene expression in neighbor similar to bacterial quorum sensing [25]. At present, the communication between artificial cells is limited to one or two signaling pathways and is much simpler compared to communication between natural cells.

### 3.3 Artificial cells as bioreactors for biomolecule fabrication

The application of artificial cells as bioreactors has the potential to enable precise execution of chemical reactions with maximum atomic utilization, and reduce the reaction activation energy as well as side reactions in specific ways [144]. In addition, the components of artificial cells are designed to avoid toxic side effects caused by drug leakage and target deletion [145]. Artificial cells as bioreactors, combined with biological enzyme catalysis can be accurately designed to simulate a series of biochemical tandem reactions, cascade reactions or some complex biochemical reactions.

Artificial cells can be a model system that has DNA/RNA replication abilities, and synthesis of protein and intracellular substances [[146], [147], [148], [149]]. For example, Tsuji and co-workers designed the phospholipid vesicles containing nucleoside triphosphate (NTPs) and RNA-replicase to enable sustainable proliferation and RNA replication by the modified freeze-thaw method [27]. The amount of RNA increased by 3-fold (326 nM) after 1 h of replication in artificial cells. A microreactor system using GUVs, which contained reverse transcription polymerase chain reaction (RT-PCR) mixture (primers, reverse transcriptase, template RNA, deoxy-ribonucleoside triphosphate, DNA polymerase and Taqman probe) was developed for the amplification of cDNA by RT-PCR and RNA detection using the fluorescent Taqman probe 6-carboxyfluorescein (FAM) [150]. Flow cytometry analysis demonstrated that 10–100 copies of amplified mRNA and rRNA in the total RNA (2 ng/μL) could be obtained.

**Fig. 8:**
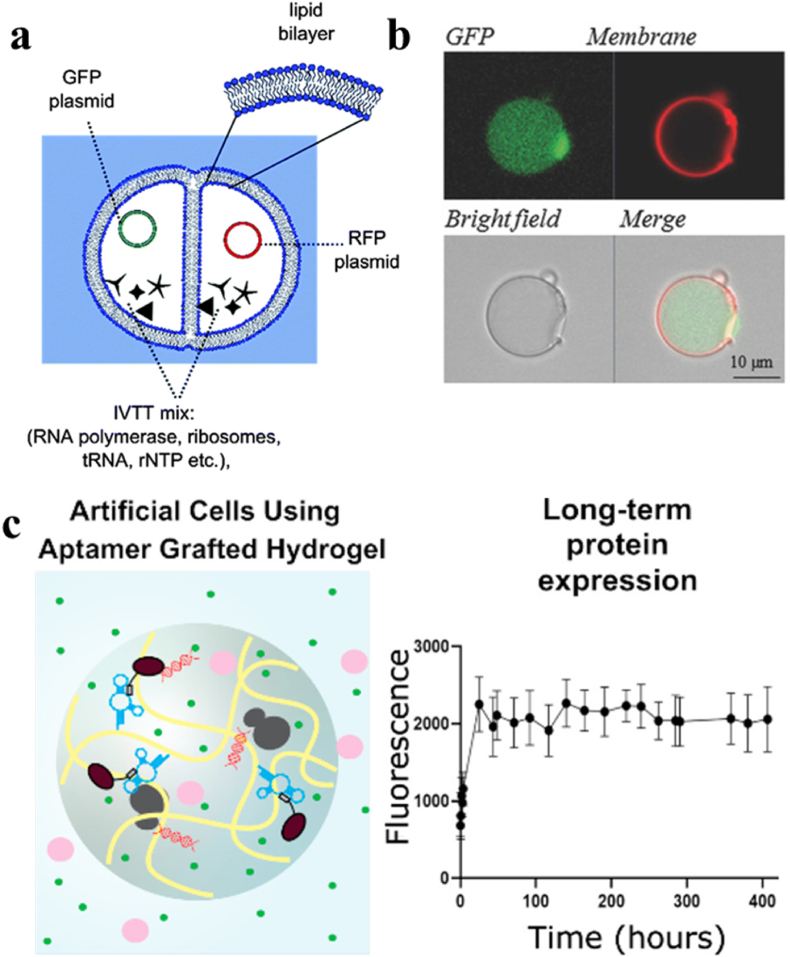
(a) Schematic diagram of artificial cells containing two compartment lipid vesicles, equipped with IVTT mixture for protein synthesis [28]. (b) Artificial cells with labeled liposome membrane by Rhodamine (red) and synthesize sfGFP (green) in liposome [106]. (c) Schematic diagram of artificial cells using aptamer grafted polymer hydrogel (left). The fluorescence expression of artificial cells (right) [152].

Another application of artificial cells as biochemical microreactors is protein synthesis within individual multi-compartments [17,28,106]. Ces and coworkers constructed artificial cells containing two-compartment lipid vesicles equipped with the *in vitro* transcription and translation (IVTT) mixture for protein synthesis (Fig. 8a). Two different proteins (green fluorescent protein (GFP) and red fluorescent protein (RFP)) were produced separately in each compartment [28]. This work provided a cell-like organization that could synthesize different proteins in different compartments for the first time. Ces and co-workers also generated three-compartment vesicles within which three-step enzymatic reactions were conducted [109]. The first compartment produced d-glucose through lactose hydrolysis. The second compartment produced H_2_O_2_ by oxidation of glucose. The third compartment synthesized fluorescent resorufin by oxidizing amplex red with H_2_O_2_. In addition, cell-free protein synthesis (CFPS) liposome-based artificial cells were established to produce therapeutic proteins (*Pseudomonas* exotoxin A (PE)) in tumors (Fig. 8b) [106]. Artificial cells containing the molecular machinery for transcription and translation synthesized a variety of proteins (molecular weight up to 66 kDa) [106]. *In vivo* transplantation of artificial cells to the mammary fat pad of BALB/c mice showed significantly higher expression of apoptotic marker caspase-3 than the untreated tumors. Researchers also developed the semipermeable artificial cells by layer-by-layer (LbL) polymer assembly for protein synthesis, which could reduce the operation cost from 55% to 13% compared to the standard dialysis cell-free protein synthesis [151]. Artificial cells capable of long-lived protein synthesis (more than 400 h) could be fabricated by using anti-His-tag aptamer grafted polymer hydrogels (Fig. 8c) [152]. Despite the promising findings in the application of artificial cells as bioreactors for protein synthesis, the development in the area is still in the proof of concept stage. The protein yield in artificial cells is relatively low (in the micro-molar range), and the protein amount inside compartments represented only a small portion of the total volume. Further work is required to improve the machinery in the artificial cells to enhance the protein yield.

**Fig. 9:**
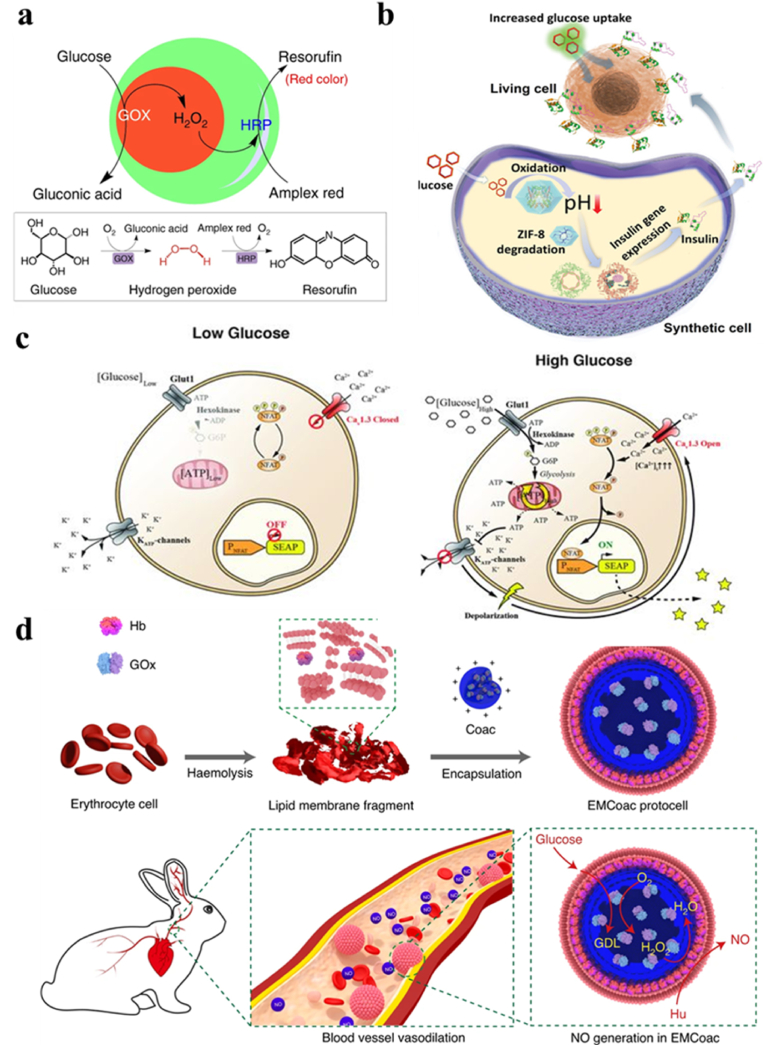
(a) Schematic illustration and reaction equations of GOX@HRP-catalyzed Amplex Red oxidation for the production of resorufin after initiation by glucose [29]. (b) Schematic diagram of artificial cells by GOx-containing zeolitic imidazolate frameworks-8 (GOx-ZIF-8) and induce living cell response [30]. (c) The diagram of glucose sensing in engineered HEK-293 cells under low and high extracellular glucose level [153]. (d) The preparation of artificial cells and the illustration of NO synthesis through cascade enzymatic reactions [154].

Artificial cells have also been used to generate substances (H_2_O_2_, NO, insulin, GLP-1, etc.) essential for other cells. Artificial cells with heterogeneous multi-compartments (PEG hydrogel containing GOx in inner compartments; poly (PEG-co-AA) hydrogel containing magnetic nanoparticles (MNP) in outer compartments) were designed (named as GOX@MNP) for cancer therapy [29]. As shown in Fig. 9a, glucose was converted into gluconic acid and H_2_O_2_ through GOx-mediated oxidation reaction inside the inner compartments. The H_2_O_2_ was then transported to the outer compartment and initiated tetramethylbenzidine oxidization mediated by MNPs. HeLa cells were effectively killed within 24 h after the treatment with a mixture of 50 mM glucose and 5 μg/mL GOX@MNP due to the cytotoxicity of hydroxyl radicals generated by the decomposition of H_2_O_2_, while the HeLa cells in the control group (5 μg/mL GOX@MNP) remained alive. This glucose-powered cancer therapeutic approach eliminated the use of toxic drugs. In addition to the application of artificial cells for the production of anti-cancer drugs, novel biocatalytic artificial cells were developed for the treatment of diabetes, which involved the integration of a glucose-sensing pseudo-organelle, a pseudo-nucleus encoding the insulin gene, and a pseudo-ribosome with a transcription and translation system within a metal-phenolic networks shell (Fig. 9b) [30]. Glucose (1.8 mg/mL) was catalyzed by GOx-containing zeolitic imidazolate frameworks-8 (GOx-ZIF-8), yielding gluconic acid, thereby reducing the pH of the solution. Consequently, ZIF-8 decomposed to release the insulin gene, and initiated insulin gene translation and synthesis within the artificial cells. Artificial β cells were also designed to regulate blood glucose concentration by constructing a glucose-inducible gene circuit, which released insulin or glucagon-like peptide I (GLP-1) (Fig. 9c) [153]. At low extracellular glucose level, the voltage-gated Cav1.3 channels was not activated. However, when the blood glucose level exceeded the threshold, the potassium channels were closed. This change in membrane potential caused the influx of Ca^2+^, leading to the secretion of insulin or GLP-1 via an internal signaling cascade, which in turn promoted glycogen synthesis, and reduced blood glucose *in vivo* [153]. Artificial cells were also designed to produce nitric oxide (NO) through cascade enzymatic reactions for the treatment of cardiovascular and other related diseases (Fig. 9d) [154]. GOx (5 mM) and hydroxyurea (2 mM) were encapsulated into the erythrocyte haemoglobin-containing membrane. H₂O₂ was produced by GOx in the presence of glucose. Hydroxyurea was then converted into NO with a yield of 8.7 μM in 180 min under the catalysis of hemoglobin, which caused vasodilation [154].

## 4 Summary and outlook

Artificial cells have been developed rapidly over the past few decades. Although remarkable progress has been made to construct complex multifunctional artificial cells, there are still significant differences between artificial cells and natural cells. The design of functional artificial cells still faces many challenges that need to be overcome. The coordination of different biochemical reactions can be modulated in order to enhance the functions of artificial cells. Further research is required to assemble multicomponent artificial cells by incorporating additional proteins. This integration is crucially important for ensuring membrane permeability and facilitating selective material exchange with the environment and adjacent natural/artificial cells. By introducing stimuli-responsive materials, it is possible to form artificial cells that can effectively respond to specific external stimuli. This advancement holds great potential for constructing controllable and tunable micro-reaction systems. Although research has demonstrated the potential of artificial cells in protein synthesis, the yield is low. It is important to achieve large-scale fabrication like normal cells in the future.

The communication between artificial cells and the extracellular environment also needs to be improved. The design of complex circuits to more realistically mimic cell growth, migration, division and genetic material replication as in living cells is appealing. The combination of machine learning and artificial intelligence approaches may effectively accelerate the production of artificial cells. The continuous improvement in the design of complex and controllable artificial cells that can simulate the real activities of cells is expected to bring the state-of-the-art technological advancement to many fields, such as drug delivery, cell therapy, regenerative medicine and biosensors.

## Declaration of competing interest

The authors declare that they have no known competing financial interests or personal relationships that could have appeared to influence the work reported in this paper.

## Data Availability

Data will be made available on request.
